# Six-legged-bound: a newly described insect gait

**DOI:** 10.1098/rsos.250143

**Published:** 2025-06-25

**Authors:** Avi Amir, O. Yuval, Amir Ayali

**Affiliations:** ^1^ School of Zoology, Faculty of Life Sciences, Tel Aviv University, Tel Aviv, Israel; ^2^ Sagol School of Neuroscience, Tel Aviv University, Tel Aviv, Israel

**Keywords:** insects, gaits, mole cricket, locomotion, in-phase synchronization

## Abstract

Locomotor behaviour is a hallmark of animal biology and ecology. Mole crickets constitute a unique group of subterranean insects that present extreme morphological and behavioural adaptations. They therefore present a valuable model for locomotion-related research. Despite their remarkable leg-morphology adaptations, mole crickets mostly demonstrate the common insect double-tripod gait for locomotion. Here we report, however, that in response to an aversive stimulus from the front, the mole cricket will consistently adopt a unique backwards gait that we have termed ‘backward-bound’. Our temporal and spatial analysis shows that this previously unreported six-legged gait comprises a cyclic alternation between the middle and hind-leg pairs with rarely observed (in insects) left–right in-phase synchronization, while the front legs display noisy and less-consistent phase dynamics. This exceptional gait is transient and is replaced by regular backwards walking after several cycles. It is employed to distance the animal quickly from danger. A gait that can be characterized as ‘forward-bound’ is also displayed by the mole cricket, albeit for a much shorter duration (up to two cycles).

## 1. Introduction

Characterizing the locomotor behaviour of animals is essential to any study of their biology and ecology (e.g. [1,2]). Locomotor behaviour constitutes the way in which the animal navigates its environment, i.e. the trajectory of the body within the environment and its interaction with the substrate; as well as the controlled and coordinated way in which the animal moves its body and appendages against each other.

**Figure 1.**
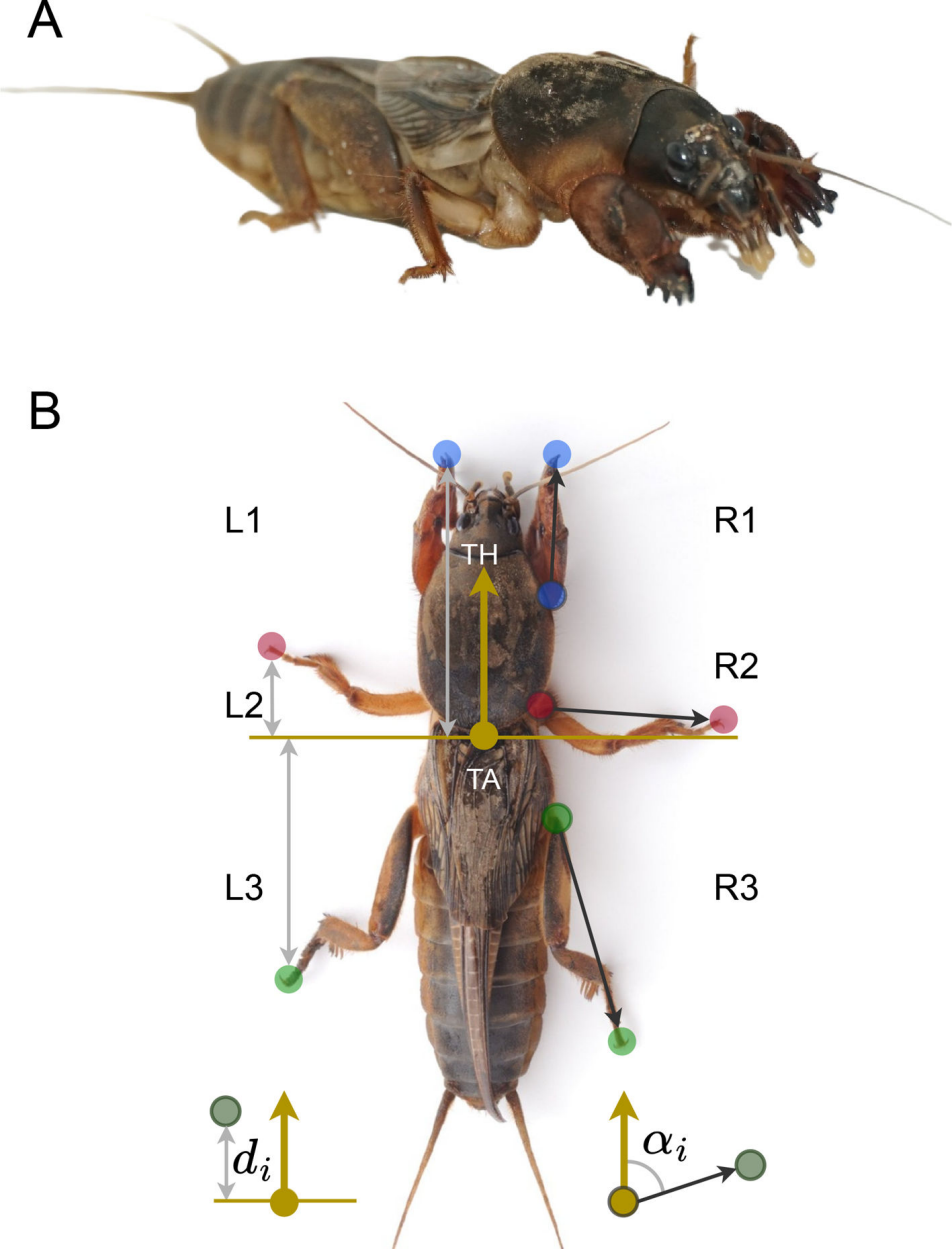
*(*A) The mole cricket presents extreme morphological adaptations to its subterranean life. The most prominent ones are the front fossorial legs and the strong, developed hind legs. Photo credit: Keren Levy. (B) Parameters used in the mole cricket gait analysis. Coloured dots depict base and tip of the three leg pairs. TA and TH: thorax–abdomen and thorax–head junctions, used to calculate leg position (*d_i_*; grey arrows, shown for left legs only) and leg angles (*a_i_*; black arrows, shown only for right legs). See §2 for details.

These different aspects of locomotion are of special interest in the case of the mole cricket (Orthoptera: Gryllotalpidae). Mole crickets are subterranean insects and among the few families of insects known to possess fossorial (digging) front legs (figure 1A). The enlarged dactyl claws on the insect’s tibia, common to all species of mole crickets, enable their unique soil-dwelling, subterranean life history. The specialized hind legs (figure 1A), in addition to serving as jumping legs (as in many other orthopteran insects), provide most of the thrust needed for pushing the animal forward when digging underground [3]. Other modifications related to the mole crickets’ life in an underground network of tunnels and burrows are their cylindrical, sclerotized body, and a pointed head (e.g. [3–5]; figure 1). Interestingly, some of these unique morphological modifications have already been distinguished in early related species preserved in mid-Cretaceous Burmese amber [6,7].

Six-legged locomotion is exceptionally effective, making insects (together with their other traits) one of the most successful groups of organisms. One reason for this pre-eminence is the insects’ remarkable ability for dynamic stability: insects can rapidly generate adaptable movement in changing environments, employing multi-level adaptations, including adaptive control mechanisms [8–10]. The dominant and ubiquitously observed insect gait is the double-tripod gait, characterized by distinctive phasing among the legs in terms of time and position: the front and rear legs on one side of the body move in phase with the middle leg on the other side and in anti-phase with their contralateral partners, resulting in two alternating tripods. The double-tripod gait is considered extremely stable and is assumed to be partly responsible for the outstanding fast locomotion of some insects and their ability to negotiate different terrains. Other common six-leg gaits include the tetrapod gait, in which four legs remain on the ground while two move at any given time; and the metachronal gait, in which the legs show sequential movement. The choice of gait often depends on speed, terrain and context.

Hence, one important aspect of dynamic and adaptable locomotion is manifested in movement-related decision-making, i.e. in selecting the gait most appropriate to the context. For example, in many animals, rapid, intense locomotion is often seen as part of their aversive response to threatening stimuli [11,12]. This type of behaviour is mostly transient and is characterized by sudden, explosive movements allowing the animal to quickly distance itself from the potential danger. Its complex and challenging environment makes the mole cricket a good model system also for the study of distinct, context-specific gait selection.

Here, we present a never-before reported insect gait that we term ‘backward-bound’, transiently demonstrated by the mole cricket in response to aversive stimuli. This unique gait is dominated by cycles of alternation between the mid-leg and hind-leg pairs (hind legs leading during backward locomotion), with intra-pair in-phase synchronization. Remarkably, such a temporal sequence has not been observed in any other mode of six-legged locomotion.

## 2. Material and methods

### 2.1. Animal maintenance and experimental procedure

Mole crickets (*Gryllotalpa tali*) were collected from the countryside around Tel Aviv, Israel, at various life stages. Insects were maintained in darkness, at room temperature (20–25°C), each in a separate 750 ml glass jar filled with autoclaved plant soil, and fed with flour beetle larvae, grass roots and thin slices of carrot. A moist environment was maintained by sprinkling each jar with 100 ml of water every 3 days. Adult males and females that were acclimatized to the laboratory conditions for at least three months were used for the experiments.

The imaging set-up comprised a 30*L* × 2.5*W* × 10*H* cm tunnel, with a 10 × 10 × 10 cm acclimatization chamber at either end. A millimetre graph paper was glued to the floor of the tunnel in order to extract a scaling factor to convert from pixels to real length units. The temperature during experiments was maintained at 24 ± 1°C. Video sequences were captured from *ca* 20 cm above the set-up, using a digital high-speed camera (500 fps; MIKROTRON motion-Blitz cube4MGE-CM4, Germany), fitted with a VS-H1218-IRC/11 lens (Vital Vision Technology Pte Ltd, Singapore). The lighting used to illuminate the set-up was covered with red cellophane to avoid glare and reduce stress to the insects.

Insects were introduced individually into one of the acclimatization chambers, and their locomotion was recorded for up to 10 min while they moved freely (spontaneously) in the tunnel section. In order to induce the bound gait, mild aversive stimuli were applied to the head or the tip of the abdomen in the form of a transient gentle push with a piece of sponge on a wooden stick. Data for the backward-bound gait comprised 20 individuals, including 10 males and 10 females (one trial per individual). Data for the forward-bound gait comprised 10 trials (8 individuals; 4 males and 4 females).

### 2.2. Video analysis and extraction of locomotion parameters

To extract kinematic parameters from the recorded data, key points on the animal’s body were tracked in all videos (figure 1B). These comprised leg tips (tip of the tarsus), leg bases (thorax-coxa junction) and the thorax–head (TH) and thorax–abdomen (TA) junctions (used to define the main body axis). To this end, frames were selected from videos of different gaits, body poses and animals, and the above noted key points were manually labelled in order to train a ResNet50 model. The trained model was then applied to the entire video dataset to detect those points in all frames (*n* = 5036). Two hundred and thirty frames (4.6%) had to be manually corrected, mostly due to cases of self-occlusion or soil particles that were attached to the body. Labelling, training and tracking were conducted using DeepLabCut (a software package for animal pose estimation [13]).

The position of the tip of a leg was defined as the shortest distance of the tip of the tarsus from the secondary body axis (perpendicular to the main body axis, with its origin at the TA point; figure 1B). Leg tip position over time, *d*_*i*_(*t*), was used to calculate swing-stance matrices and inter-leg phases. Using the maxima and minima in this signal for each leg, forward stances were defined as the ranges from the anterior-most to the posterior-most leg tip positions, while swing was defined as the rest of the signal. Backward stances were defined in the opposite way (i.e. from the posterior-most to the anterior-most leg tip positions). The inter-leg phase of leg *i* and *j* was calculated by computing the cross-correlation between *d*_*i*_(*t*) and *d*_*j*_(*t*).

Leg angle was defined as the angle between the vector connecting the leg base to its tip, and the main body axis (figure 1B). To obtain the average leg angle, the circular mean was first used to average leg angles over time, and then across videos. Similarly, the circular s.d. was used to obtain the s.d. of leg angles over time, and then averaged across videos using a regular mean. Statistics for the mean leg angle were calculated using the Kuiper’s test for circular data. Statistics for the s.d. were calculated using the two-sample *t*‐test.

## 3. Results

**Figure 2.**
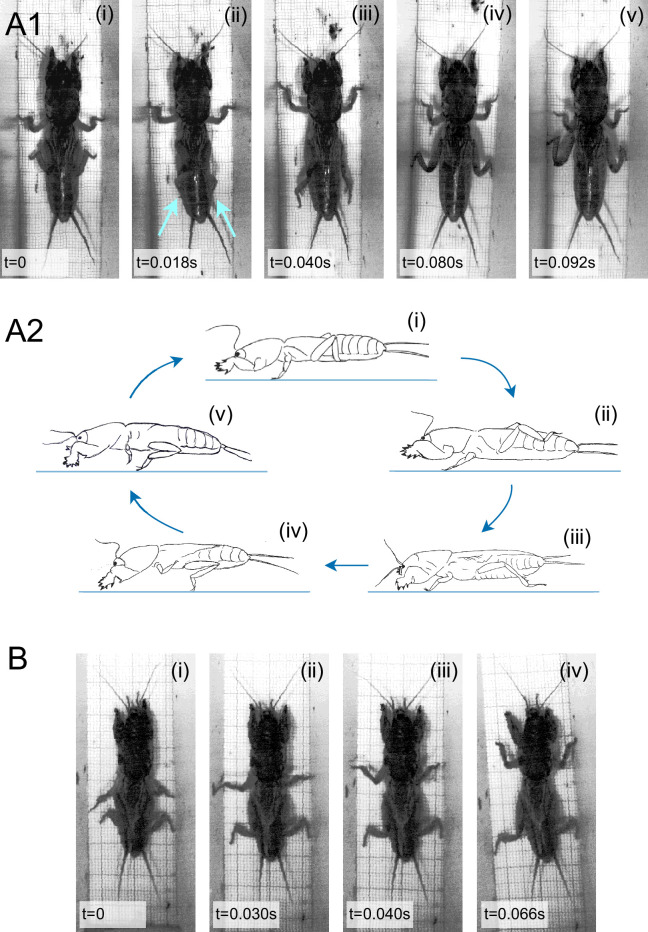
The unique mole cricket bound gait: (A1) Sample images extracted from a video used in the presented analysis of the backward-bound gait, highlighting the left–right legs’ synchronous symmetric movements. Arrows depicts hind-leg tips; and (A2) An artist’s side view (based on a video) of the backward-bound gait, demonstrating the leg and body trajectories. (B) Four main stages in the forward-bound gait (*cf*. A).

Mole crickets in our experimental set-up demonstrated mostly forward walking with occasional backward locomotion, both while exhibiting the characteristic double-tripod gait (see also [5,14,15,]). Upon experiencing an aversive stimulus from the front (in the shape of an approaching decoy comprising a piece of sponge on a wooden stick), whether standing still or during a forward locomotion bout, the insect swiftly switched to moving rapidly backwards, adopting a unique mode of leg coordination that we have termed ‘backward-bound’ (figure 2A). Experiments in which the insect was successfully induced to demonstrate the backward-bound gait were included in the subsequent analysis if the motion was consistent for at least three complete cycles. Out of 41 adult mole crickets in our colony, 20 insects demonstrated such uninterrupted bound gait for up to six cycles before switching to double-tripod backward walking. Ten to twelve cycles were also (rarely) observed (not among the analysed experiments).

**Figure 3.**
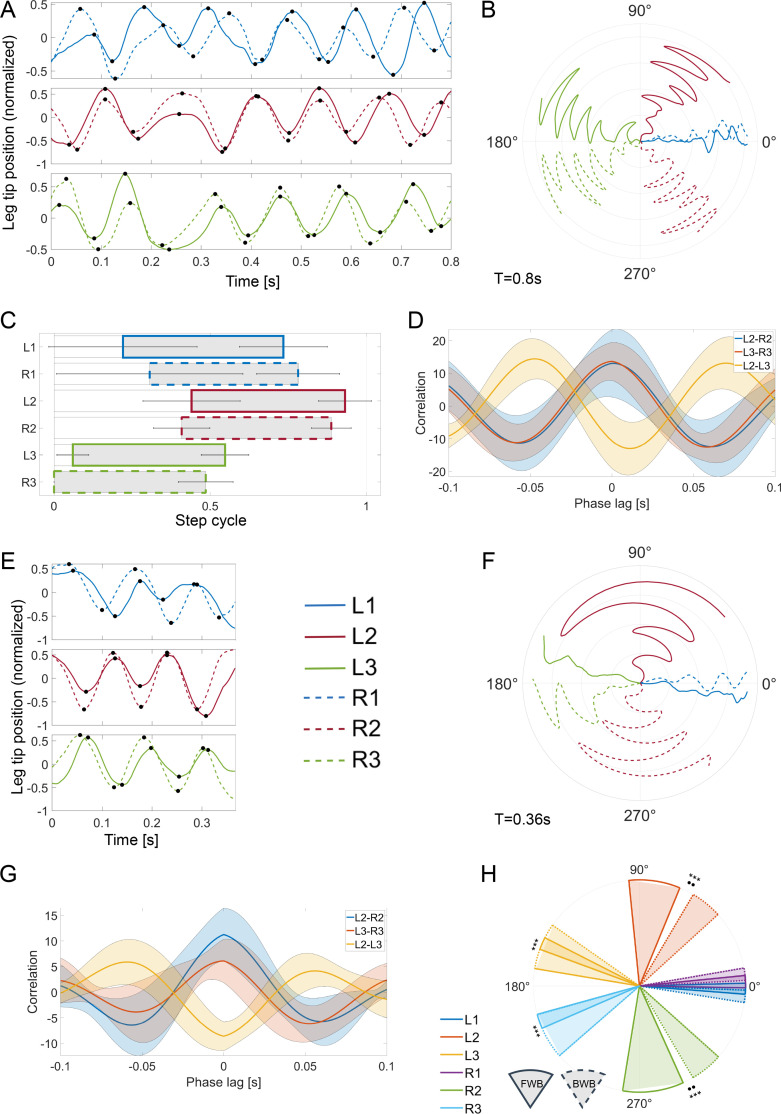
Temporal and spatial characteristics of the mole cricket bound gait. (A) An example of the leg-tip positions over time during backward-bound gait. Colours as in figure 1B; solid lines: left legs; dashed lines: right legs. Dots denote the peaks of the step cycle, corresponding to the anterior (maximum) and posterior (minimum) positions of the leg tips relative to the insect’s body. (B) An example of the leg angles during backward-bound (see A and §2; same example as in A). Distance from the origin depicts time. (C) Phasing of all legs relative to the right hind leg during backward-bound. Coloured bars show the average phase of the stance in normalized step cycle coordinates (extracted from the leg tips’ position data; see black dots in A), with s.d. (*n* = 20). (D) Inter-leg phases (mean ± s.d.) during the backward-bound gait as demonstrated by cross-correlation of leg pairs. (E) An example of the leg-tip positions in time during forward-bound (*ca* three cycles), cf*.* A. (F) Leg angles during the forward-bound, cf. B. (G) Inter-leg phases (mean ± s.d.) during the forward-bound gait as demonstrated by cross-correlation of leg pairs. cf. D. (H) Legs’ angular coverage in forward- versus backward-bound. Circular sectors show mean ± s.d. of leg angles. Analysis based on data similar to that shown in B and F. Solid lines: forward-bound (*n* = 10); dotted lines: backward-bound (*N* = 20). Differences in the mean angle, **p < 0.005; differences in the s.d., ***p < 0.0005.

Utilizing video tracking and machine learning, we analysed the details of the legs’ movements during backward-bound, as well as the distinct phasing between the legs (figures 2A and 3A–D, *n* = 20; 10 males and 10 females). This unique gait is characterized by left–right in-phase synchronization between leg pairs, with the two dominant leg pairs—the middle and the hind legs—consistently alternating (figure 3A,C, electronic supplementary material, video S1): i.e. while the middle legs are in the stance (pushing against the ground), the hind legs are in swing (raised and moved far back, figures 2A and 3A–D). Conversely, while the hind legs are in stance, the middle legs are in swing. Figure 3B complements the example shown in figure 3A by offering a description of the spatial trajectories of all legs during a backward-bound gait sequence (the angle between the vector connecting the leg base to its tip and the main body axis; backwards—movement direction—is to the left; time is denoted by distance from the origin). The data demonstrate once again the left–right symmetry within leg pairs. The front pair of legs is relatively somewhat irregular in timing and phase, as evident from the large variation depicted in the front leg data in figure 3C. As suggested by figure 2A2, the insect’s abdomen is raised as its hind legs push against the ground, which, in a side view, gives the impression that the mole cricket is bouncing backwards.

Presenting a standing mole cricket with an aversive stimulus from behind, i.e. touching its rear parts with a similar decoy stimulus, sometimes resulted in a brief and transient (up to three cycles) ‘forward-bound’ gait (figures 2B and 3E,F, *n* = 10). A comparison of leg angles reveals significant differences in the trajectory of the middle and hind legs in forward- versus backward-bound (figure 3H; note the changed colour code for clarity): in forward-bound the middle legs span a larger angular sector and are oriented more perpendicularly to the body (in a more posterior position), while the hind legs show altogether very limited angular motion. The cross-correlation analysis presented in figure 3D,G suggests overall similar phase relations to those described above for the backward-bound gait. Thus, despite similar temporal characteristics, the spatial analysis suggests that different legs take a dominant role in thrust generation in forward- versus backward-bound.

The role of the bound gait appears to be to quickly distance the insect from the aversive stimulus’s source before switching to the more stable and controlled double-tripod gait. However, within the current study, we did not conduct a detailed comparative analysis of the different mole cricket locomotion gaits (see [15]).

## 4. Discussion

Mole crickets present an intriguing model system for the study of insect locomotion. Their cryptic lifestyle in the subterranean environment greatly restricts our knowledge regarding their physiology and behaviour. Their extreme morphological modifications, and specifically their leg adaptations, make any prediction regarding their modes of locomotion and walking gaits challenging. Based on the extreme and diverse adaptations seen in the different leg pairs, it is somewhat surprising that mole crickets predominately use the common double-tripod gait for walking [5,14,15]. However, it may be less surprising to have discovered that these fascinating insects present a unique, previously undescribed locomotion gait—the six-legged-bound gait.

Gait selection is a specific case of motor pattern selection [16,17]. This concept is based on the accepted understanding that, in vertebrates and invertebrates alike, most locomotion-related motor patterns are controlled by central nervous system neuronal networks known as central pattern generators (CPGs). CPG-generated rhythmic output induces an alternation between antagonistic motor neuron groups, resulting in motor behaviours with distinct phase relationships. Research in various invertebrate and vertebrate models has revealed common design principles of motor selection and decision-making: namely context-dependent selection of the appropriate motor network and the distinct network’s motor output (distinct phase scheme).

The double-tripod gait is prevalent among all insect models studied thus far: from the slow walking stick insect, where it is mostly observed in young immature animals, to the fast cockroach, where the double-tripod gait is the principal gait used in practically all walking speeds and environmental contexts [10]. Similarly, it was found in moth [18], as well as in the fly [19]. The major feature of the double-tripod gait is alternation within left–right leg pairs. This is a dominant trait of all six-legged gaits (as well as in most legged animals). Indeed, left–right in-phase motion is seldom seen in other stable insect walking gaits.

Hence, within the above framework, the bound gait, characterized by left–right in-phase synchronization of all leg pairs, is unique. It may thus reflect a specific functional architecture (functional connectivity) of the mole cricket’s locomotion CPGs. The left–right in-phase synchronization is expressed in a distinct descending neuronal (brain) and neuro-modulatory context—in response to an aversive stimulus. Furthermore, previous findings suggest that the bound gait constitutes an unstable state of the CPG networks that control locomotion in insects (see [16,20] for a discussion of network stability in insect locomotion). Future work will focus on the distinct conditions and mechanisms that enable the emergence of these fundamentally unique phase relations and coupling between the unit pattern-generator circuits controlling the different legs during bound (for a discussion of coupling in insect locomotion see [21,22]).

Animal escape responses are by definition fast and robust, comprising in principle a burst of muscle activation [11,12]. Widely studied examples include the crayfish tail-flip behaviour (e.g. [23,24]) and the escape jump of the locust [25,26]. Escape responses, however, are not necessarily simple. They are the result of complex sensorimotor processes that include the processing of the input, the decision, the generation of motor commands and the activation of muscles that move the animal and maximizes its chances of survival [11,27]. This is well exemplified by the much-studied cockroach escape behaviour [28,29]. The herein described forward-bound bears some similarity to the evasive jump of the locust (as also seen in other orthopterans). However, in contrast to a jump that relies only on the hind legs [25], the forward-bound gait involves the alternation of at least the middle and hind pairs of legs, and is therefore very different and more complex. Though not common, mole crickets do jump or hop on occasion using their hind legs only. Similar to the case of the locust, in the adult mole cricket the jump will precede flight initiation (Amir & Ayali, unpublished). The backward-bound, while clearly an evasive response, needs to be examined under a different framework, as it comprises a longer sequence of controlled leg movements demonstrating a unique pattern of coordination (compared with other locomotion gaits). The backward-bound seems particularly suited to escape from an aversive stimulus in a tunnelling insect like the mole cricket.

The novel bound gait described herein may be somewhat reminiscent of the previously reported galloping gait of the dung beetle [30], as the galloping beetle also demonstrates a very unusual left–right legs’ synchrony. The case of the mole cricket is more conspicuous, however, for several major reasons, among them is the fact that the bound is one of several gaits available to the mole cricket, to be transiently selected at the appropriate context. This presents, of course, major challenges in control, as well as in biomechanics. Even more significant is the fact that unlike in the case of the beetle’s galloping (where the hind legs are dragged behind the insect without use), the bound gait involves all six legs of the insect, including the very dominant pair of hind legs.

The current study is part of a major endeavour aimed at constructing a detailed comparative description of the multiple locomotion gaits of the mole cricket (e.g. [15]). Such a comprehensive description is required in order to fully comprehend the six-legged-bound gait within the full scope of locomotion behaviours of this intriguing insect.

## Data Availability

All raw data that served for generating the presented figures can be found in the supplementary file (electronic supplementary material, table S1) [31]. Further data as required may be available upon request.
